# Environmental Chemicals: Integrative Approach to Human Biomonitoring and Health Effects

**DOI:** 10.3390/toxics10060314

**Published:** 2022-06-10

**Authors:** Virgínia Cruz Fernandes, Diogo Pestana

**Affiliations:** 1 REQUIMTE/LAQV, Instituto Superior de Engenharia do Porto, Instituto Politécnico do Porto, Rua Dr. António Bernardino de Almeida 431, 4249-015 Porto, Portugal; 2 CINTESIS & NOVA Medical School | Faculdade de Ciências Médicas da Universidade Nova de Lisboa, Campo Mártires da Pátria 130, 1169-056 Lisboa, Portugal

In recent decades, citizen awareness of toxic chemicals has been a topic of interest, particularly concerning national and international policy decision makers, expert/scientific platforms, and health protection organizations (WHO, UNEP, CDC, EFSA, IPEN, etc.). Even in a world of quick information access, synthesizing crucial scientific knowledge and evidence about environmental exposure and related health problems into readily understandable concepts and statistics remains a remarkable challenge.

Throughout life, people are exposed to both naturally occurring and human-made chemicals. These exposures are a root cause of a significant disease burden that could be prevented by reducing or removing chemical exposure. According to the WHO: in total, more than 2 million deaths and 53 million disability-adjusted life years (DALYs) were attributable to environmental exposure and management of selected chemicals, a higher estimate compared with those in 2016 and 2012 [1]. The largest contributors were cardiovascular diseases (42%, 848,778 deaths), chronic obstructive pulmonary disease (COPD, 26%, 517,734 deaths) and cancers (17%, 333,867 deaths). However, only a small number of chemical exposures, among the many chemicals we are exposed to, are considered in these analyses [1].

People are exposed to a wide range of environmental chemicals in their daily lives, in different contexts, and via multiple routes, including indoors and outdoors (e.g., air, soil, and water contamination; consumer products (e.g., cosmetics, cleaning agents, textiles, food, etc.); industrial chemicals; etc.) [2,3,4,5,6,7]. From this extensive exposure by several routes, the multiple contaminants to which we are exposed is exhausting and worrying. Some examples of the most reported toxic chemicals are pesticides [8,9,10,11], heavy metals [12] polycyclic aromatic hydrocarbon (PAH) [13,14], polychlorinated biphenyls (PCB) [15], pharmaceuticals [16], plastic-related chemicals (e.g., flame retardants, phthalates, etc.) [17,18], and microplastics [19,20,21]. Currently, it is impossible to escape exposure to environmental chemicals, namely those with endocrine-altering potential (endocrine-disrupting chemicals, EDCs).

Unintended exposure to pesticides can be extremely hazardous to humans and other living organisms as they are designed to be poisonous. Pesticide exposure is linked with various diseases including cancer, asthma, dermatitis, endocrine disorders, reproductive dysfunctions, immunotoxicity, neurobehavioral disorders, and congenital defects [22,23,24]. Data from a number of PAH occupational health studies suggest that there is an association between lung cancer and exposure to PAH compounds [25]. Studies in human and animals suggest a correlation between flame retardants exposure and adverse health outcomes, namely thyroid disorders; neurobehavior and development disorders; and reproductive, immunological, metabolic, oncological, and cardiovascular diseases [17,26]. Phthalate exposures were associated with all-cause and cardiovascular mortality, with societal costs approximating USD 39 billion/year or more in the USA [27]. Recently, microplastics that may cause inflammatory lesions, originating from the potential of their surface to interact with the tissues, have been reported. In addition, the increasing incidence of neurodegenerative diseases, immune disorders, and cancers may also be related to the increased exposure microplastics and their co-contaminants [19]. The effects of exposure in human health are influenced not only by the type and concentration of the chemicals but also by the effects and complexity of mixtures and, more importantly, by the timing of exposure. Indeed, there is an increased vulnerability to chemical exposure in windows of greater susceptibility, especially during childhood and pregnancy, which may impair lifetime health. Therefore, there is a need to biomonitor and evaluate all exposures across lifespans and its interaction with our own unique characteristics, the ‘exposome’.

As a complex field, researchers continue to wrestle with important issues, which requires an integrative and multidisciplinary research approach to this problematic, resorting to complementary methodologies to measure human exposure to environmental chemicals and to assess their health effects. One can define three main pillars: (1) environmental chemical analysis and development of new detection methods, with the identification and quantification of biomarkers of exposure and/or effect and/or susceptibility and development of new analytical methodologies for the detection of biomarkers in several human matrices (e.g., blood, plasma, serum, urine, and adipose tissue); (2) evaluation of biological effects, through the assessment of exposure impact on human health (e.g., general population, and people with obesity or diabetes) and/or resorting to experimental and mechanistic approaches (in vitro/in vivo models); and (3) data management and statistical analysis, namely in study design and sampling in the human population.

Biomonitoring studies are a good example of this complementarity, encompassing the measurement of internal levels of chemicals/metabolites in easily accessible biological fluids or tissues, and aiming to understand environmental health threats and to assist policy measures, namely in susceptible populations such as children. It requires analytical methods of high selectivity and high sensitivity due to low concentrations and limited sample volumes. Toxic chemicals cover a wide range of chemical groups with different physical–chemical properties. Therefore, scientific literature presents several analytical methods even for the same substance groups. Depending on the chemical group, the human biomonitoring biomarkers are either parent compounds or metabolites. A large variety of matrices have been analyzed (blood, urine, adipose tissue, hair, nails, breast milk, etc.). This complexity calls for the urgent need to carry out further studies on the appropriate analytical methods for each group of compounds and matrices. Biomonitoring studies identify new chemicals in human tissues, monitor the distribution of exposures among the general population, and provide a measure of potential health risk.

Preventing diseases arising from chemical environments requires the development of a consistent and rational approach to human biomonitoring as a complementary tool to assist in providing evidence-based public health and environmental measures, confirming the health effects of toxic chemical exposures, and validating regulatory actions and policies.
